# Preoperative Assessment to Predict Difficult Airway Using Multiple Screening Tests

**DOI:** 10.7759/cureus.46868

**Published:** 2023-10-11

**Authors:** Dhwani N Trambadia, Payal Yadav, Sargunaraj A

**Affiliations:** 1 Anesthesiology, Pandit Deendayal Upadhyay Medical Hospital, Rajkot, IND; 2 Anaesthesiology, Chirayu Private Hospital, Jaipur, IND; 3 Anaesthesiology, Sri Manakula Vinayagar Medical College and Hospital, Puducherry, IND

**Keywords:** atlantooccipital, interincisor gap, lemon score, upper lip bite, mallampatti, difficult, predictors, intubation, assesssment, airway

## Abstract

Background

Predicting a difficult airway is one of the necessities in anesthesiology practice. Recognition of an obviously difficult airway leads to a series of communication and preparations to assist, as well as the establishment and maintenance of the airway. In this study, we compared various predictors of difficult laryngoscopy/intubation to determine the best possible difficult airway predictors. The present study aimed to evaluate the sensitivity, specificity, positive predictive value (PPV), negative predictive value (NPV), and accuracy of the following airway assessment tests to predict difficult airway: (1) Modified Mallamapati test; (2) thyromental distance; (3) inter-incisor gap; (4) upper lip bite test; (5) LEMON airway assessment test; and (6) atlantooccipital movement.

Methodology

A total of 300 patients who presented for different operative procedures were selected. Screening tests were done in the preoperative examination room. The tests included the Modified Mallamapati test, thyromental distance, upper lip bite test, inter-incisor gap, LEMON airway assessment, and atlantooccipital movement. Laryngoscopy was done in the operation theater and the view was classified according to Cormack-Lehane’s scale. Using this clinical data, the sensitivity, specificity, PPV, and NPV of each test in predicting difficult airways were calculated.

Results

The thyromental distance test had the highest sensitivity, NPV, and accuracy. The upper lip bite test had the highest specificity and PPV. LEMON airway assessment test had the lowest specificity, PPV, NPV, and accuracy. Thyromental distance had the highest accuracy followed by the Modified Mallampati test. Inter-incisor gap had low sensitivity and PPV, and the atlantooccipital extension test had low sensitivity.

Conclusions

The currently available screening tests for difficult intubation have only poor-to-moderate discriminative power when used alone. No single airway test can provide a high index of sensitivity and specificity for the prediction of difficult airways. The upper lip bite test had the highest specificity and the thyromental distance test had the highest NPV. Every anesthesiologist must be trained and equipped to deal with now much less common, unexpected failure to intubate.

## Introduction

Anesthesia is a unique specialty. Some airways may be difficult to maintain under mask anesthesia but are easily intubated, other airways are difficult to intubate but may be maintained with mask anesthesia for the duration of the operation, and some are difficult to manage in both aspects [1]. One of the fundamental responsibilities of the anesthesiologist is to mitigate the adverse effects of anesthesia on the respiratory system by maintaining airway patency and ensuring adequate ventilation and oxygenation [2]. Accurate assessment of the airway can prevent catastrophic perioperative events such as hypoxia, hypercapnia, arrhythmia, and cardiac arrest. Successful airway management requires a range of knowledge and skill sets, specifically the ability to predict difficulty with airway management and formulate an airway management plan, as well as the skills necessary to execute the plan using a wide array of available airway devices. Recognition of an obviously difficult airway leads to a series of communication and preparations to assist, as well as the establishment and maintenance of the airway. Respiratory events are the common causes of anesthesia-related morbidity, out of which 85% are related to mistakes regarding airway management resulting in permanent cerebral damage due to hypoxia and 30% of anesthesia-related deaths. This makes it the most important cause of major anesthesia-related morbidity and mortality. There are many tests to predict difficult laryngoscopy and intubation. Mallamapati et al. introduced a screening test that classifies the visibility of the oropharynx. Patil Aldreti measured the distance of the thyroid notch to the mentum and the distance of the manubrium sterni to the mentum, that is, thyromental distance and sternomental distance. A recently introduced test is the upper lip bite test which assesses the ability of the patient to cover the mucosa of the upper lip bite with lower incisors. Other tests include inter-incisor gap, LEMON airway assessment, and the atlantooccipital movement test. These tests have been tested in various previous studies for the prediction of difficult airways but the combination of tests included here is unique. Therefore, we conducted this study to compare the sensitivity, specificity, positive predictive value (PPV), negative predictive value (NPV), and accuracy of these tests as methods of airway assessment for difficult laryngoscopy.

## Materials and methods

After obtaining institutional ethical committee approval, 300 patients with the American Society of Anesthesiologists Physical Status I-III and aged between 18 and 65 years for different operative procedures were selected between January 2019 and November 2019.

Inclusion and exclusion criteria

Inclusion criteria included ASA Physical Status I-III, patients posted for elective surgeries and scheduled to receive general anesthesia (orthopedic, ENT, ophthalmologic, abdominal, urologic, and gynecological procedures), and patients aged between 18 and 65 years.

Exclusion criteria included ASA Physical Status IV-V; uncooperative and unwilling patients not following verbal commands; patients with a history of burns, trauma, or surgeries to the airway; patients with tumors or masses in the neck or the airway; patients with restricted mobility at the neck and mandible; patients with an inability to sit; edentulous patients; or those who needed awake intubation.

Screening tests

Multiple screening tests were performed in the preoperative examination room including the Modified Mallamapati test, thyromental distance, upper lip bite test, inter-incisor gap, LEMON airway assessment, and atlantooccipital movement test.

Modified Mallamapati Test

Sampson and Young’s modification of the Mallamapati test recorded oropharyngeal structures visible upon maximal mouth opening. Each patient when seated was asked to open their mouth maximally and protrude the tongue without phonation. The view was classified as Class I (visualization of the soft palate, hard palate uvula, and anterior and posterior tonsillar pillars), Class II (visualization of the soft palate, hard palate, and uvula), Class III (visualization of the soft and hard palate and the base of the uvula), and Class IV (only hard palate is visible).

Thyromental Distance

The distance between the tip of the thyroid cartilage to the tip of the inside of the mentum is measured in the neck fully extended position. Grade 1: >6.5 cm implies easy laryngoscopy and intubation; Grade 2: -6.0 cm to 6.5 cm implies difficult intubation but possible; and Grade 3: <6 cm implies that intubation may not be possible.

Upper Lip Bite Test

This is performed by assessing the ability of the patient to cover the mucosa of the upper lip with lower incisors. This test is rated as Class I if the lower incisors can bite the upper lip above the vermilion line, Class II if the lower incisors can bite the upper lip below the vermilion line, and Class III if the lower incisors cannot bite the upper lip.

Inter-Incisor Gap

It is defined as the distance between the incisors (or alveolar marginal) with the mouth opening maximally. Grade 1: >4 cm and Grade 2: <4 cm.

LEMON Airway Assessment

The score with a maximum of 10 points is calculated by assigning 1 point for each of the following criteria: L - look externally: Facial trauma, large incisors, beard, mustache, or large tongue. E - evaluate the 3-3-2 rule Incisor distance: -3 finger, hyoid mental distance: -3 finger, thyroid to floor of mouth distance: -2 finger. Mallamapati score - >3. O - obstruction: epiglottitis, peritonsillar abscess, and trauma. N - neck mobility (limited neck mobility).

Atlantooccipital Movement

In this test, the patient is asked to hold the head erect, facing directly to the front. Subsequently, they are asked to extend the head maximally and the examiner estimates the angle traversed by the occlusal surface of the upper teeth. Grade 1: >35, Grade 2: 22-34, Grade 3: 12-22, Grade 4: <12.

In the operation theater, the monitors attached included non-invasive blood pressure, electrocardiogram, pulse oximeter, and end-tidal CO_2_. Patients were preoxygenated with 100% oxygen for four deep breaths for 30 seconds using an oxygen flow of 6 L/minute. After premedication, induction of anesthesia was done with an appropriate inducing agent followed by a depolarizing muscle relaxant. Laryngoscopy was done and the view was classified according to Cormack-Lehane’s scale, without any external laryngeal manipulation. This scale is graded as Grade 1 (vocal cords visible), Grade 2 (only posterior commissure visible), Grade 3 (only epiglottis visible), and Grade 4 (none of the above visible). Difficult visualization was described as Grade 3 and 4 classification. Easy visualization was described as Class 1 and 2 classification. Confirmation of intubation was done by bilateral auscultation of lung fields and capnography.

Failed intubation was defined as not being able to intubate the patient’s airway and the need for surgical airway manipulation. Easy intubation was defined as being able to intubate without bougie/stylet and without any external larynx manipulation.

## Results

A total of 300 patients scheduled to receive general anesthesia and endotracheal intubation were selected. Of the 300 patients, 179 (60%) were male and 121 (40%) were female. As shown in Table 1, 110 (36%) patients belonged to the ASA I grade, 103 (34%) to the ASA II grade, and 87 (29%) to the ASA III grade.

**Table 1:** Distribution of the patients among different ASA grades. ASA = American Society of Anesthesiology

Grade	Number of patients	Percentage
ASA I	110	36%
ASA II	103	34%
ASA III	87	29%
Total	300	100%

Table 2 shows the distribution of the patients among the different classes of the Modified Mallampati classification. There were 250 (214 + 36) patients in classes I and II, predicted as easy intubation. Whereas 50 (41+9) patients belonged to classes III and IV, predicted as difficult intubation.

**Table 2:** Distribution of patients among different classes of Modified Mallampatti classification. MMT = Modified Mallampatti test

MMT	Number of patients	Percentage
Class I	214	71%
Class II	36	12%
Class III	41	13%
Class IV	9	3%
Total	300	100%

The number of patients in each class of thyromental distance is presented in Table 3. In total, 230 cases were included in Class 1, which predicted easy intubation. However, there were 50 patients in Class II and 20 patients in Class III, which predicted difficult intubation.

**Table 3:** Distribution of patients among different classes of the thyromental test.

Thyromental test	Number of patients	Percentage
Class I	230	85%
Class II	50	11%
Class III	20	3%
Total	300	100%

The number of patients in each class of inter-incisor gap is presented in Table 4. In total, 230 patients were included in Grade 1, which predicted easy intubation, and 70 patients were included in Grade 2, which predicted difficult intubation.

**Table 4:** Distribution of patients among two grades of the inter-incisor gap test.

Inter-incisor gap	Number of patients	Percentage
Grade 1	230	76%
Grade 2	70	23%
total	300	100

The number of patients in each class of the upper lip bite test is presented in Table 5. In total, 290 patients belonged to Class I and Class II, which predicted easy intubation, and 10 patients belonged to Class III, which predicted difficult intubation.

**Table 5:** Distribution of patients among different classes of the upper lip bite test (ULBT).

ULBT	Number of patients	Percentage
Class I	200	66%
Class II	90	30%
Class III	10	3%
Total	300	100%

The number of patients in each class of LEMON airway assessment is given in Table 6. A total of 230 patients had LEMON scores of 0/10, which predicted easy intubation, and 70 patients had LEMON scores >0/10, which predicted difficult intubation.

**Table 6:** Distribution of patients among two different scores of the LEMON airway assessment test.

LEMON airway assessment	Number of patients	Percentage
Score 0/10	230	76%
Score >0/10	70	23%
Total	300	100

The number of patients in each class of the atlantooccipital test is presented in Table 7. A total of 280 patients belonged to atlantooccipital extension grades I and II, which predicted easy intubation, and 20 patients belonged to grades III and IV, which predicted difficult intubation.

**Table 7:** Distribution of patients among different grades of the atlantooccipital test.

Atlantooccipital extension	Number of patients	Percentage
Grade I	250	83%
Grade II	30	10%
Grade III	15	5%
Grade IV	5	1.6%
Total	300	100%

The number of patients in each grade of Cormack-Lehane classification of glottis exposure is shown in Table 8. A total of 264 patients belonged to Cormack-Lehane grades I and II, indicating easy intubation, and 36 patients belonged to grades III and IV, indicating difficult intubation.

**Table 8:** Distribution of patients among different Cormack-Lehane grades.

Cormack-Lehane grade	Number of patients	Percentage
Grade I	234	78%
Grade II	30	10%
Grade III	32	10.6%
Grade IV	4	1.3%
Total	300	100%

The sensitivity, specificity, PPV, and NPV of each preoperative test are mentioned in Table 9. P-values <0.05 were considered significant.

**Table 9:** Standard formulas to compare different tests for data analysis. Sensitivity = (a)/(a + c) Specificity = (d)/(b + d) Positive predictive value = (a)/(a + b) Negative predictive value = (d)/(c + d) Accuracy = (a + d)/(a + b + c + d)

	Difficult	Easy	Total
Predicted difficult	True positive (a)	False positive (b)	(a + b)
Predicted easy	False negative (c)	True negative (d)	(c + d)
Total	(a + c)	(b + d)	(a + b + c + d)

The distribution of various predictive tests based on Cormack-Lehane laryngoscopy grading is demonstrated in Table 10.

**Table 10:** Distribution of various predictive tests based on Cormack-Lehane laryngoscopy grading. MMT = Modified Mallampati test; TMD = thyromental distance; IIG = inter-incisor gap; ULBT = upper lip bite test; AOE = atlantooccipital test; CL = Cormack-Lehane classification

Factors	Grade	Total number of cases	CL I and II	CL III and IV
MMT	Easy	250	242	8
	Difficult	50	22	28
TMD	Easy	255	249	6
	Difficult	45	15	30
IIG	Easy	230	204	26
	Difficult	70	60	10
ULBT	Easy	290	262	28
	Difficult	10	2	8
LEMON score	Easy	230	204	26
	Difficult	70	60	10
AOE	Easy	280	254	26
	Difficult	20	10	10

**Figure 1:**
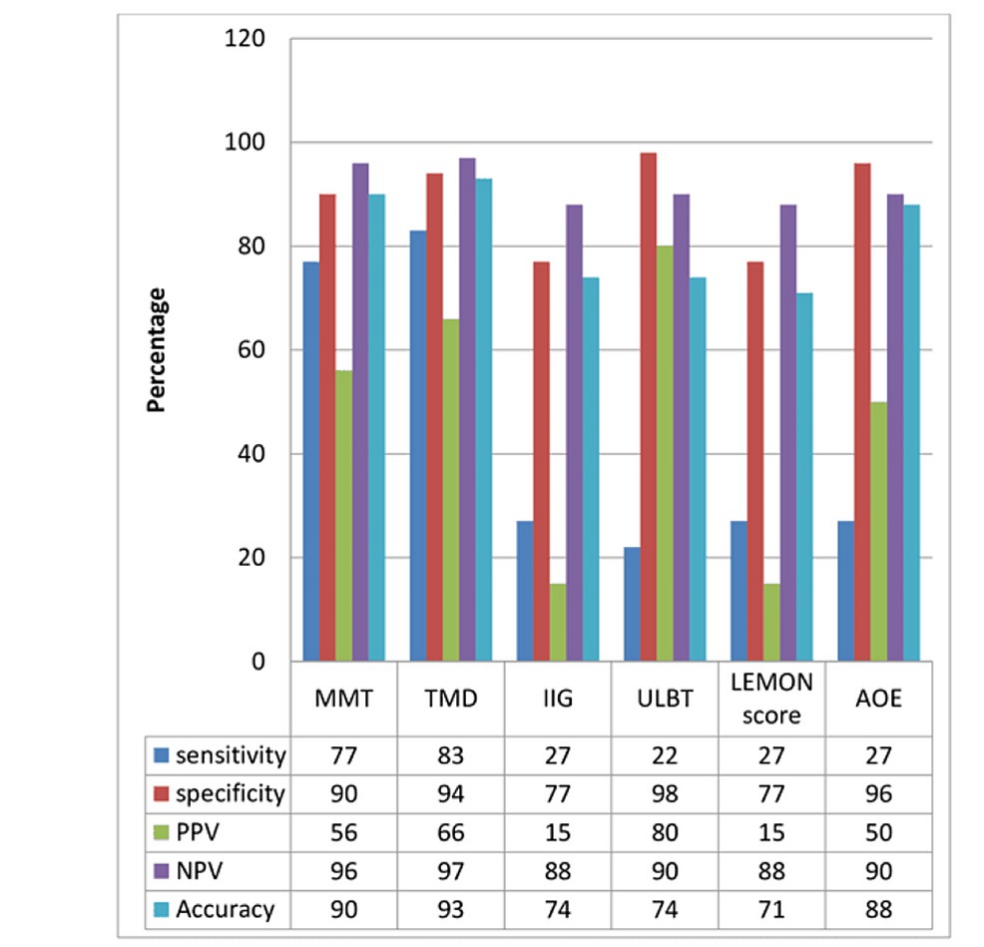
Comparison of various predictive tests. MMT = Modified Mallampati test; TMD = thyromental distance; IIG = inter-incisor gap; ULBT = upper lip bite test; AOE = atlantooccipital test; PPV = positive predictive value; NPV = negative predictive value

A comparison of various predictive tests including sensitivity, specificity, PPV, NPV, and accuracy is demonstrated in Figure 1. The highest sensitivity, NPV, and accuracy were observed with the thyromental distance test. The upper lip bite test had the highest specificity. Inter-incisor gap and LEMON score had the lowest PPV.

## Discussion

Unanticipated difficult laryngoscopic tracheal intubation remains a primary concern among anesthesiologists. Predicting difficult intubation can significantly reduce anesthesia-associated morbidity and mortality [1]. The recorded incidence of difficult laryngoscopy and tracheal intubation is 0.1-13% in general anesthesia [2], with intubation failure occurring in 0.05-0.35% of cases [3]. An ASA closed claims analysis of adverse outcomes associated with anesthesia showed that the most common cause of serious injury was inadequate ventilation and esophageal intubation. Difficult tracheal intubation tests to predict difficult intubation should have high sensitivity to identify most patients in whom intubation would be difficult. It should also have a high PPV so that only a few patients who can be actually intubated easily are subjected to the protocol for the management of difficult intubation [4]. The incidence of difficult intubation in our study was 12% and the failed intubation incidence was 1.3%. In our study, we found that the thyromental distance test has the highest sensitivity, NPV, and accuracy. The upper lip bite test had the highest specificity and PPV. The LEMON airway assessment test had the lowest specificity, PPV, NPV, and accuracy. Thyromental distance had the highest accuracy followed by the Modified Mallampatti test. Inter-incisor gap had low sensitivity and PPV, and the atlantooccipital extension test had low sensitivity.

The demographic characteristics in this study were comparable to the studies by Krobbuaban et al. [5], Shing et al. [6], Leopold et al. [7], Suvarna et al. [8], Krobubban et al. [9], Khan et al. [10], and Huh et al. [11]. In contrast to the present study, we found high sensitivity, specificity, and NPV with low PPV for the Modified Mallampati test in the studies by Khan et al. [12], Safavi et al. [13], and Shah et al. [14]. The present study was comparable to the studies by Domi [15] and Bilgin et al. [16] on the Mallamapati score. In contrast to this study, the studies done by Domi [15] and Bilgin et al. [16] showed low sensitivity, low PPV, high specificity, and high NPV. In our study, thyromental distance showed the highest sensitivity and highest NPV among all the tests (Modified Mallampati test, inter-incisor gap, upper lip bite test, LEMON score, and atlantooccipital tests). We found very low sensitivity, low PPV, and acceptable specificity and NPV. we found high sensitivity, high specificity, acceptable PPV, and high NPV. This study was comparable with the study by Yildiz et al. [17] and Vasvani et al. [18].

This study was comparable with the study of Domi [15] and Yildiz et al. [17] which showed very low sensitivity and low PPV for inter-incisor gap test. In contrast to this study, the study by Vasvani et al. [18] showed acceptable sensitivity and PPV for the upper lip bite test. This test showed the highest specificity and PPV among all the tests included in this study.

Vasvani et al. [18] reported a significant correlation between old age, obesity, and high body mass index with the incidence of difficult intubation. They showed acceptable sensitivity, high specificity, high PPV, and high NPV. The upper lip bite test showed the least sensitivity among all tests. This study showed good specificity and NPV, which was comparable with another study that showed that an airway assessment score based on the LEMON method can successfully stratify the risk of intubation in the emergency department. This study was comparable with the study by Rao et al. [19] which showed low sensitivity and high specificity and NPV. However, in contrast to this study, it showed a low PPV.

Of all the tests, the LEMON airway assessment score had the least specificity, PPV, and NPV. Limited neck extension hinders the proper alignment of three axes making laryngoscopy and intubation difficult. It was measured during visual assessment in our study. The present study showed the least sensitivity and excellent specificity and NPV. In contrast to this study, Vasvani et al. [18] showed no significant correlation (p > 0.77).

Limitations

This study had a few limitations. Most laryngoscopies and intubations were performed by undergraduate students, which might have contributed to a higher number of difficult laryngoscopies. Airway management may not follow standard guidelines, or there may be interpersonal variations in the anesthetic management in terms of experience, preparation, and availability of equipment for intubation which may also have an impact on the number of difficult laryngoscopies and intubations. Finally, there is a lack of standardized cut-off values for the preoperative airway parameters. Different authors have used different cut-off values for preoperative tests which can impose some difficulties in comparing different findings.

## Conclusions

The currently available screening tests for difficult intubation have only poor-to-moderate discriminative power when used alone. No single airway test can provide a high index of sensitivity and specificity for the prediction of a difficult airway. The upper lip bite test had the highest specificity and the thyromental distance test had the highest NPV for predicting difficult intubation. Every anesthesiologist must be trained and equipped to deal with, now much less common, unexpected failure to intubate.

## References

[1] Arné J, Descoins P, Fusciardi J, Ingrand P, Ferrier B, Boudigues D, Ariès J (1998). Preoperative assessment for difficult intubation in general and ENT surgery: predictive value of a clinical multivariate risk index. Br J Anaesth.

[2] Benumof JL (1991). Management of the difficult adult airway. With special emphasis on awake tracheal intubation. Anesthesiology.

[3] Wilson ME, Spiegelhalter D, Robertson JA, Lesser P (1988). Predicting difficult intubation. Br J Anaesth.

[4] Savva D (1994). Prediction of difficult tracheal intubation. Br J Anaesth.

[5] Krobbuaban B, Diregpoke S, Kumkeaw S, Tanomsat M (2005). The predictive value of the height ratio and thyromental distance: four predictive tests for difficult laryngoscopy. Anesth Analg.

[6] Shiga T, Wajima Z, Inoue T, Sakamoto A (2005). Predicting difficult intubation in apparently normal patients: a meta-analysis of bedside screening test performance. Anesthesiology.

[7] Eberhart LH, Arndt C, Cierpka T, Schwanekamp J, Wulf H, Putzke C (2005). The reliability and validity of the upper lip bite test compared with the Mallampati classification to predict difficult laryngoscopy: an external prospective evaluation. Anesth Analg.

[8] Kaniyil S, Anandan K, Thomas S (2018). Ratio of height to thyromental distance as a predictor of difficult laryngoscopy: a prospective observational study. J Anaesthesiol Clin Pharmacol.

[9] Krobbuaban B, Diregpoke S, Kumkeaw S (2006). An assessment of the ratio of height to thyromental distance compared to thyromental distance as a predictive test for prediction of difficult tracheal intubation in Thai patients. J Med Assoc Thai.

[10] Khan ZH, Mohammadi M, Rasouli MR, Farrokhnia F, Khan RH (2009). The diagnostic value of the upper lip bite test combined with sternomental distance, thyromental distance, and interincisor distance for prediction of easy laryngoscopy and intubation: a prospective study. Anesth Analg.

[11] Huh J, Shin HY, Kim SH, Yoon TK, Kim DK (2009). Diagnostic predictor of difficult laryngoscopy: the hyomental distance ratio. Anesth Analg.

[12] Khan ZH, Maleki A, Makarem J, Mohammadi M, Khan RH, Zandieh A (2011). A comparison of the upper lip bite test with hyomental/thyrosternal distances and mandible length in predicting difficulty in intubation: a prospective study. Indian J Anaesth.

[13] Safavi M, Honarmand A, Zare N (2011). A comparison of the ratio of patient's height to thyromental distance with the modified Mallampati and the upper lip bite test in predicting difficult laryngoscopy. Saudi J Anaesth.

[14] Shah PJ, Dubey KP, Yadav JP (2013). Predictive value of upper lip bite test and ratio of height to thyromental distance compared to other multivariate airway assessment tests for difficult laryngoscopy in apparently normal patients. J Anaesthesiol Clin Pharmacol.

[15] Domi R (2009). A comparison of Wilson sum score and combination Mallampati, tiromental and sternomental distances for predicting difficult intubation. Macedonian J Med Sci.

[16] Bilgin H, Ozyurt G (1998). Screening tests for predicting difficult intubation. A clinical assessment in Turkish patients. Anaesth Intensive Care.

[17] Yildiz TS, Korkmaz F, Solak M (2007). Prediction of difficult tracheal intubation in Turkish patients: a multi-center methodological study. Eur J Anaesthesiol.

[18] Vaswani JP, Wadhwa S (2022). Comparison of predictive value of upper lip bite test ratio of height to thyromental distance and Mallampati classification for the anticipation of difficult intubation in apparently normal patients. J Res Innov Anesth.

[19] Rao KV, Dhatchinamoorthi D, Nandhakumar A, Selvarajan N, Akula HR, Thiruvenkatarajan V (2018). Validity of thyromental height test as a predictor of difficult laryngoscopy: A prospective evaluation comparing modified Mallampati score, interincisor gap, thyromental distance, neck circumference, and neck extension. Indian J Anaesth.

